# On Two Numerical Techniques for Light Scattering by Dielectric Agglomerated Structures

**DOI:** 10.6028/jres.098.046

**Published:** 1993

**Authors:** Akhlesh Lakhtakia, George W. Mulholland

**Affiliations:** Department of Engineering Science and Mechanics, Pennsylvania State University, University Park, PA 16802; National Institute of Standards and Technology, Gaithersburg, MD 20899-0001

**Keywords:** agglomerates, coupled dipolc method, light scattering, method of moments, smoke

## Abstract

Smoke agglomerates are made of many soot sphcres, and their light scattering response is of interest in fire research. The numerical techniques chiefly used for theoretical scattering studies are the method of moments and the coupled dipole moment. The two methods have been obtained in this tutorial paper directly from the monochromatic Maxwell curl equations and shown to be equivalent. The effects of the finite size of the primary spheres have been numerically delineated.

## 1. Introduction

Electromagnetic scattering problems involving complicated geometries are treated numerically nowadays. Apart from some low- or high-frequency methods [1, 2] and the T-matrix method [3], implementation of most numerical techniques entails a partitioning of the region occupied by the scatterer into may subregions. This is generally true whether a differential formulation is used or an integro differential formalism.

The method of moments (MOM) [4–6], as applied to an inhomogeneous dielectric scatterer, is an approach based on the evaluation of an electric field volume integral equation over the region occupied by the scatterer. This region is partitioned into a number of subregions; the electric field in each subregion is represented by a subregional basis function; and the volume integral equation is converted into a set of simultaneous algebraic equations that are solved using standard procedures. The subregions are generally cubes, although the relevant self-terms are usually evaluated as that of equivoluminal spheres.

Whereas the MOM is an *actual field* formalism, the coupled dipole method (CDM) is based on the concept of an *exciting field*. The CDM was formulated intuitively in 1969 by Purcell and Pennypacker [7] for dielectric scatterers, although it had by that time a rich history spanning many decades [8]. Conceived from a microscopic viewpoint, the CDM has only a semi-microscopic basis; indeed, the operational basis for applying the CDM to boundary value problems is totally macroscopic [9]. Both the MOM and the CDM were recently extended to bianisotropic scatterers and their respective *weak* and *strong* forms were shown to be equivalent [10].

This tutorial paper contains a complete derivation of the MOM and the CDM for the scattering of time-harmonic electromagnetic waves by an inhomogeneous dielectric object possessing an isotropic permittivity, the starting point of the exercise being the monochromatic Maxwell curl equations. A central topic of the paper is the elucidation of the relationship between the MOM, which is widely used in electrical engineering, and the CDM, which is motivated by concepts in atomic physics [7]. Our treatment is directed towards the researcher who is interested in understanding these methods but who may not be a specialist in electromagnetic field theory.

A current topic of considerable interest is light scattering by agglomerated structures made up of individual spheres arranged in a low-density structure. Examples of such structures include smoke agglomerates formed in fires or internal combustion engines; various materials produced in combustion systems including silica and titanium dioxide; and, perhaps, interstellar dust. The earliest relevant analyses [11, 12] of light scattering from smoke were based on the Rayleigh-Debye approximation, in which the field exciting any particular primary sphere is taken to be just the field that is actually incident on the agglomerate. Such a procedure neglects multiple scattering effects, and is acceptable for primary spheres with size parameter (= radius multiplied by the free space wavenumber) less than about 0.2. The typical size parameters for the smoke agglomerates mentioned above lie in the range 0.1 to about 0.25 at visible frequencies.

Both the CDM [13, 14] and the MOM [15, 16] have been applied to compute scattering from smoke agglomerates in the recent past, since neither technique suffers from the limitations of the Rayleigh-Debye approximation. While the MOM and the CDM are expected to give the same results for infinitesimally small size parameters [17], the methods – as ordinarily used – do not yield identical results as the primary sphere size increases [18, 19]. This is because the CDM has been used chiefly in what may be called its *weak* form, while it is the *strong* form of the MOM that is in standard usage [10]. The strong form is valid for larger primary spheres because the effect of the singularity of the free space dyadic Green’s function is better estimated therein than in the weak form. This becomes clearer in the following sections.

## 2. Volume Integral Equation

**Fig. 1:**
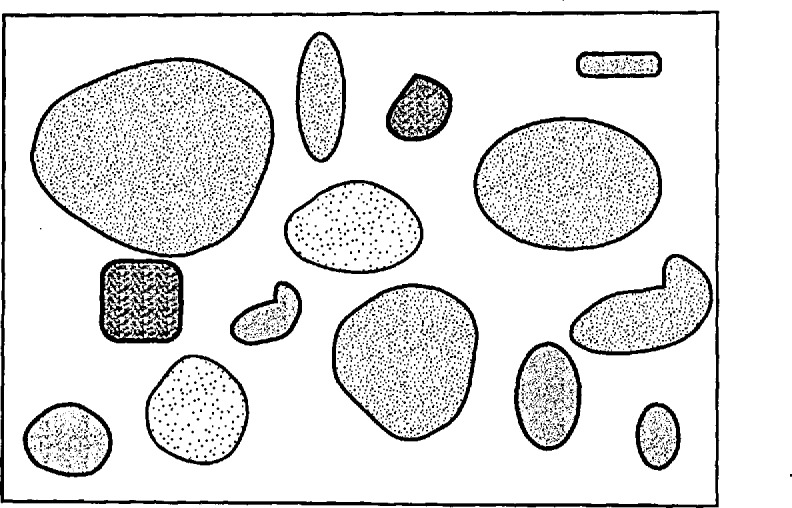
Schematic of the scattering problem. The unshaded region *V*_ext_ extends to infinity in all directions, while the shaded regions collectively constitutc *V*_int_.

As is schematically illustrated in Fig. 1, let all space be divided into two mutually disjoint regions, *V*_int_ and *V*_ext_, that are distinguishable from each other by the occupancy of matter. The region *V*_ext_ is vacuous; hence,

$$D(x)=\epsilon_{0}E(x);x\in V_{\text{ext}.}$$ (1a)

$$B(x)=\mu_{0}H(x);x\in V_{\text{ext}}.$$ (1b)

The region *V*_int_ is filled with an isotropic, linear, possibly inhomogeneous, dielectric continuum with frequency dependent [exp(−*iωt*)] constitutive equations

$$D(x)=\epsilon_{0}\;\epsilon_{r}(x)\;E(x);x\in V_{\mathrm{int}},$$ (2a)

$$B(x)=\mu_{0}\;H(x);x\in V_{\mathrm{int}},$$ (2b)

where *ϵ*_r_(***x***) is the complex relative permittivity scalar. The square root of *ϵ*_r_(***x***) is the local complex refractive index, Imag [*ωϵ*_0_*ϵ*_r_(***x***)] is the local conductivity, and *ω* is the circular frequency.

There is no requirement that *V*_int_ be a simply-connected convex region. Sharp corners and cusps should be absent, it being preferable that the boundary surface that separates *V*_int_ from *V*_ext_ be at least once-differentiable everywhere to enable the unambiguous prescription of a unit normal at every point on it. Furthermore, the maximum linear extent of *V*_int_ must be bounded so that only the region *V*_ext_ extends out to infinity in all directions.

The monochromatic Maxwell curl equations, in the absence of any externally impressed sources, are given in *V*_ext_ as

$$\nabla \times E(x)-i\omega \mu_{0}H(x)=0;x\in V_{\text{ext},}$$ (3a)

$$\nabla \times H(x)-i\omega \epsilon_{0}E(x)=0;x\in V_{\text{ext},}$$ (3b)

with *0* denoting the null vector. Similarly, in *V*_int_ we have

$$\nabla \times E(x)-i\omega \mu_{0}H(x)=0;x\in V_{\text{int},}$$ (4a)

$$\nabla \times H(x)+i\omega \epsilon_{0}\epsilon_{r}(x)E(x)=0;x\in V_{\mathrm{int}.}$$ (4b)

On rewriting Eq. (4b) as

$$\begin{array}{c} \nabla \times H(x)+i\omega \epsilon_{0}E(x)+i\omega \epsilon_{0}[\epsilon_{r}(x)-1]\;E(x)\\ =0;x\in V_{\mathrm{int},} \end{array}$$ (4c)

and comparing it with Eq. (3b), we are able to state the Maxwell curl equations *everywhere* as

$$\nabla \times E(x)-i\omega \mu_{0}H(x)=0;x\in V_{\mathrm{int}}+V_{\text{ext},}$$ (5a)

$$\nabla \times H(x)+i\omega \epsilon_{0}E(x)=J(x);x\in V_{\mathrm{int}}+V_{\text{ext}.}$$ (5b)

In Eq. (5b), the volume electric current density ***J***(***x***) ≡ ***0*** for ***x*** ∈ *V*_ext_, but

$$J(x)=-i\omega \epsilon_{0}\;[\epsilon_{r}(x)-1]\;E(x);x\in V_{\mathrm{int}.}$$ (5c)

In this fashion a volume electric current density has effectively replaced the dielectric matter occupying the region *V*_int_ [20, Sec. 3–11].

The volume electric current density ***J***(***x***) defined by Eq. (5c) is not a fictitious entity in the present context. We must remember that, while the region *V*_ext_ is vacuous, the region *V*_int_ is occupied by dielectric matter. The monochromatic polarization current density in this dielectric matter is given by

$$\begin{array}{c} J_{\text{pol}}(x)=-i\omega \epsilon_{0}\{\text{Real}[\epsilon_{r}(x)]-1\}\;E(x);\\ x\in V_{\mathrm{int},} \end{array}$$ (6a)

while the conduction current density is given by

$$J_{\text{cond}}(x)=\omega \epsilon_{0}\text{Imag}[\epsilon_{r}(x)]\;E(x);x\in V_{\mathrm{int}.}$$ (6b)

It is now easy to see that

$$J(x)=J_{\text{pol}}(x)+J_{\text{cond}}(x);x\in V_{\mathrm{int},}$$ (6c)

which implies that ***J***(***x***) is not merely a mathematically convenient quantity for dielectric scatterers.

It is enough that we look for the solution of the differential equations (5a, b) in terms of only the electric field, since the magnetic field everywhere may be obtained from Eq. (5a) if the electric field is known everywhere: ***H***(***x***) = ∇ *×* ***E***(***x***)*/iωμ*_0_. On taking the curl of both sides of Eq. (5a), and then substituting for ∇ *×* ***H***(***x***) from Eq. (5b) in the result, we get

$$\begin{array}{c} \nabla \times \nabla \times E(x)-{k_{0}}^{2}E(x)=i\omega \mu_{0}J(x);\\ x\in V_{\mathrm{int}}+V_{\text{ext},} \end{array}$$ (7)

where *k*_0_ = *ω*(*μ*_0_*ϵ*_0_)^1/2^ is the free space wavenumber. We take cognizance of the fact that when ***J***(***x***) is set equal to zero everywhere, Eq. (7) reduces to the celebrated vector Helmholtz equation.

As shown in Appendix A, it follows from Eq. (7) that the electric field ***E***(***x***) is the solution of the volume integral equation

$$\begin{array}{l} E(x)-E_{\text{inc}}(x)=i\omega \mu_{0}{∭}_{\nu_{\mathrm{int}}}d^{3}x^{\prime }\\ \{G(x,x^{\prime })\cdot J(x^{\prime })\}x\in V_{\mathrm{int}}+V_{\text{ext},} \end{array}$$ (8a)

or, equivalently,

$$\begin{array}{l} E(x)-E_{\text{inc}}(x)={k_{0}}^{2}{∭}_{\nu_{\mathrm{int}}}d^{3}x^{\prime }\{[\epsilon_{r}(x^{\prime })-1]\\ G(x,x^{\prime })\cdot E(x^{\prime })\};x\in V_{\mathrm{int}}+V_{\text{ext},} \end{array}$$ (8b)

on using Eq. (5c). Here

$$\begin{array}{c} G(x,x^{\prime })=[I+(1/{k_{0}}^{2})\nabla \nabla ][\exp(ik_{0}\left|x-x^{\prime }\right|)/\\ 4\pi \left|x-x^{\prime }\right|] \end{array}$$ (8c)

is the free space dyadic Green’s function and **I** is the identity dyadic. The field ***E***_inc_(***x***) is the solution of Eq. (7) when ***J*(*x*)** ≡ **0** for all ***x*** ∈ *V*_int_ *+ V*_ext_, and is the electric field existing in *V*_int_ *+ V*_ext_ if *ϵ*_r_(***x***) = 1 for all ***x*** ∈ *V*_int_. The standard radiation conditions are satisfied by the right sides of Eqs. (8a, b) as *k*_0_|***x***|→∞ [21].

## 3. Intermediate Remarks

Equation (8a) is utilized in setting up the CDM, while the integral Eq. (8b) is solved in the MOM, and it is this commonality that partially begets the algorithmic equivalence of the two techniques. In setting up the solutions of Eq. (7) in the form of the integral Eqs. (8a, b), we have brought in two significant topics that need some rumination right now: (i) dyadics and (ii) the free space dyadic Green’s function.

Dyadics are as American as apple pie, being the brainchildren of Josiah Willard Gibbs. In 1884, Gibbs circulated a pamphlet introducing the concept and nomenclature of dyadics. Mathematics books with dyadic notation were written every so often earlier in this century, but most mathematicians appear to have eventually discarded dyadics in favor of tensors. In electromagnetics, though, dyadic notation has been used with great profit, the books by van Bladel [22], Fedorov [23] and Chen [24] being immensely popular. A short exposition on dyadics was brought out by Lindell [25] in 1981, and is much recommended to the interested reader.

A dyadic serves as a linear mapping from one vector to another vector; thus, a dyadic **D** is a mapping from ***a*** to ***b*** given by ***b* = D** · ***a***. This property leads to the idea of a *dyad* that is *composed* of two vectors, i.e., **D_12_** = ***d*_1_*d*_2_**. It follows that **D_12_** · ***a =d*_1_(*d*_2_** · ***a***) and ***a*** · **D_12_ = (*a*** · ***d*_1_**) ***d*_2_** are vectors, and **D_12_ × *a* = *d*_1_(*d*_2_ × *a***) and ***a ×* D_12_ = (*a* × *d*_1_)*d*_2_** are dyads, and the oft-used appellation *bivectors* for dyads appears justified [26]. Because a dyadic can be written as the sum of dyads, the general representation of a dyadic is the sum **D = *Σ****_km_* ***D****_km_****d****_k_****d****_m_* [27].

The identity dyadic **I** is such that **D** · **I = D = I** · **D**, and the null dyadic **0** is such that **D** · **0 = 0 = 0** · **D.** The simplest antisymmetric dyadic is ***u ×* I**, where ***u*** is any vector of unit length. Even vector differential operators can be thought of as dyadics; thus, the curl operator is written as ∇ × **I**, and the divergence operator as ∇ · **I**, in dyadic notation. It is not possible for us to go through all the wonderful properties of dyadics than can be exploited in theoretical electromagnetics research, so we refer the interested reader to the compendiums in the books by Chen [24], van Bladel [22], and Varadan et al. [28]. The main feature of computational significance is that, as dyads are bivectors, all dyadics used in this paper can be thought of as 3 × 3 matrices.

The second issue concerns the singularity of the dyadic Green’s function **G(*x***, ***x*′**) used in Eqs. (8a, b) and defined in Eq. (8c): the factor exp (*ik*_0_ |***x****−****x*′**|)/4*π*|***x****−****x*′**| goes to infinity as |***x***−***x***′|→0. When evaluating **G(*x, x*′)**, the second derivatives therefore have to be carefully handled. As shown by van Bladel [29], the classical procedure leads one to write

$$\begin{array}{l} {∭}_{v}d^{3}x^{\prime }G(x,x^{\prime })\cdot b(x^{\prime })=-(1/3{k_{0}}^{2})b(x)+\;\\ P.V.{∭}_{v}d^{3}x^{\prime }\{G(x,x^{\prime })\cdot b(x^{\prime })\};x\in V, \end{array}$$ (9)

where P.V. stands for “principal value.” When computing the P.V. integral on the right side of Eq. (9), an infinitesimally small spherical region centered about the point ***x****′* = ***x*** is excluded from the domain of integration. Computing the P.V. integral is not problematic because [30]

$$\begin{array}{l} G(x,x^{\prime })=[(I-u_{x}u_{x})+(i/k_{0}\left|X|\right)(1+i/k_{0}\left|X\right|)\\ (I-3u_{x}u_{x})]\{\exp(ik_{0}\left|X\right|)/4\pi \left|X\right|\};\left|X\right|\neq 0, \end{array}$$ (10)

is not singular when the source point ***x***′ and the field point ***x*** do not coincide; here ***X*** = ***x*** *−* ***x***′ and ***u****_x_* = ***X*/|*X***|. Yaghjian [31] has modified the right side of (9) to an expression wherein the excluded infinitesimal region need not be spherical.

We will use an alternative approach to evaluate the integral (9), as shown in Appendix 2. In this approach [6, 10, 32], the region of integration is split into two regions: one region includes the point ***x****'* = ***x*** in its interior, while the second one is the remainder. Use is made of Gauss’ theorem and the Green’s function for Poisson’s equation to determine this integral. This procedure is attractive as it places very little restrictions on the shape and the size of the region surrounding the point ***x***′ = ***x***.

## 4. The Method of Moments

The method of moments is a general mathematical procedure for transforming a linear operator equation into a set of simultaneous algebraic equations, and the interested reader is referred to [4], Chap. 1, for a simple introduction. We are, however, confining ourselves here to the application of MOM to electromagnetic scattering problems.

Although the MOM has grown increasingly sophisticated in the last decade [6, 33], a simple version [5] suffices for the easy conversion of the integral equation (8b) into a set of simultaneous algebraic equations. We begin by partitioning the scatterer region *V*_int_ into simply connected subrogions *V_m_*, 1 *≤ m ≤ M*, each bounded by a closed surface *S_m_*, so that Eq. (8b) can be transformed to

$$\begin{array}{l} E(x)=E_{\text{inc}}(x)+{k_{0}}^{2}\sum_{m}{∭}_{V_{m}}d^{3}x^{\prime }\{[\epsilon_{r}(x^{\prime })-1]\\ G(x,x^{\prime })\cdot E(x^{\prime })\},x\in \sum_{m}V_{m}+V_{\text{ext}.} \end{array}$$ (11)

The main features of this partitioning scheme are as follows:

- Each subregion *V_m_* is modeled as being homogeneous such that
  $$\epsilon_{r}(x)=\epsilon_{r,m};x\in V_{m.}$$ (12)
- The maximum linear cross-sectional extent 2*a_m_* of *V_m_* is such that *a_m_*/λ < 0.1 and *a_m_|ϵ*_r,_*_m_*^1/2^|/*λ* < 0.1; that is, the dimension *a_m_* is no more than a tenth of the wavelength *λ* in the exterior region *V*_ext_ as well as of the wavelength in the subregion *V_m_*.
- The surface *S_m_* that bounds the subregion *V_m_* is sufficiently smooth so as to be at least once differentiable, which enables the unambiguous prescription of a normal at any point on *S_m_*.

Satisfaction of these three conditions, in practice, means that the union *Σ_m_V_m_* of the subrogions is only approximately coincident with the scatterer region *V*_int_. Condition (i) can lead to an artificial material discontinuity across the interface of two adjacent subrogions, therefore the simple MOM algorithm given in this section works best when adjacent subrogions do not differ widely in their permittivities. Condition (ii) ensures that the spatial variations of the electromagnetic field inside each subregion are small enough so that each sub-region can be thought of as a dipole scatterer [34], though different authors use somewhat different upper bounds on the subregional size [35, 36]. Condition (iii) is mostly ignored by MOM users, their usual practice being to use cubical subrogions. Thus, the adequacy and the accuracy of the MOM – and the CDM – results depend in large measure on the adequacy of the partitioning scheme.

Two ancillary aspects of the partitioning scheme need to be stated for the simple MOM algorithm. First, the incident field must have slow enough spatial variations that it may be considered almost spatially constant over any subregion. Second, not only the permittivity but also the actual field is assumed spatially constant in each subregion. Together, these two assumptions constitute a long wavelength approximation [37], whose consequences are exploited in the MOM as well as in the CDM.

Let the volume of *V_m_* be denoted by $\nu_{m}={∭}_{V_{m}}d^{3}x$, and let ***x****_m_* denote a distinguished point (such as the centroid) lying inside *V_m_*. On setting ***x = x****_k_* in Eq. (11) we get the relation

$$\begin{array}{l} E(x_{k})-{k_{0}}^{2}[\epsilon_{r,k}-1]{∭}_{V_{k}}d^{3}x^{\prime }\\ \left\{G(x_{k},x^{\prime })\cdot E(x^{\prime })\}=E_{\text{inc}}(x_{k})+{k_{0}}^{2}\sum_{m,m\neq k}[\epsilon_{r,m}-1\right]\\ {∭}_{V_{m}}d^{3}x^{\prime }\{G(x_{k},x^{\prime })\cdot E(x^{\prime })\};1\leq k\leq M. \end{array}$$ (13a)

Next, on using the approximation ***E***(***x***) ≅ ***E***(***x****_m_*) for all ***x*** ∈ *V_m_* and carrying out the integrations over all subrogions, we get

$$\begin{array}{l} E_{k}-(\epsilon_{r,k}-1)[{k_{0}}^{2}M_{k}-L_{k}]\cdot E_{k}=\\ E_{\text{inc}}(x_{k})+{k_{0}}^{2}\sum_{m,m\neq k}\{\nu_{m}[\epsilon_{r,m}-1]\\ G(x_{k,}x_{m})\cdot E_{m}\};1\leq k\leq M, \end{array}$$ (13b)

where ***E****_m_* ≅ ***E***(***x****_m_*). In obtaining Eq. (13b) from Eq. (13a), we have accomplished the integration on the subregion *V_m_*, *m ≠ k*, very simply by evaluating the specific integrand at ***x****_m_* and multiplying it by the volumetric capacity ***v****_m_*. The integral $\int \int \int_{V_{k}}d^{3}x^{\prime }G(x_{k},x^{\prime })\cdot E(x^{\prime })$, on the other hand, has been estimated using Eq. (A2-5a).

With the assistance of Eqs. (5c) and (12), we transform Eq. (13b) into 3*M* simultaneous algebraic equations compactly stated in vector dyadic notation as

$$E_{\text{inc}}(x_{k})=\sum_{m\;\in \{1,2,\ldots M\}}[A_{km}\cdot E_{m}];1\leq k\leq M,$$ (14a)

where the *3M* unknowns are the cartesian components of the fields ***E****_m_*, 1 *≤ m ≤ M*. The dyadic kernel used in Eq. (14a) is given as

$$\begin{array}{l} A_{km}=I\;\delta_{km}+(1-\epsilon_{r,k})\delta_{km}[{k_{0}}^{2}M_{k}-L_{k}]+\\ {k_{0}}^{2}\nu_{m}(1-\epsilon_{r,m})(1-\delta_{km})G_{km,} \end{array}$$ (14b)

where ***δ****_km_* is the Kronecker delta and the definition

$$\begin{array}{c} G_{km}=G(x_{k,}x_{m})=[\left(I-X_{km}X_{km}/{\left|X_{km}\right|}^{2}\right)+\\ \left(i/k_{0}\left|X_{km}\right|\right)\left(I-3X_{km}X_{km}/{\left|X_{km}\right|}^{2}\right)+\\ {\left(i/k_{0}\left|X_{km}\right|\right)}^{2}\left(I-3X_{km}X_{km}/{\left|X_{km}\right|}^{2}\right)]\\ \left[\exp\left(ik_{o}\left|X_{km}\right|\right)/4\pi \left|X_{km}\right|\right];m\neq k, \end{array}$$ (15)

follows from Eq. (A2-6a) with ***X****_km_* = ***x****_k_ −* ***x****_m_*. In this straightforward and simple version of the MOM, wherein the electric field has been assumed to be piecewise constant over the scatterer region *V*_int_, Eq. (14a) has to be solved for the three cartesian components of all ***E****_m_* for specified ***E***_inc_(***x***).

Bearing in mind that all of our dyadics can be easily thought as 3 × 3 matrices, one can solve Eq. (14a) using a variety of matrix manipulation techniques. The Gauss elimination procedure [38] is simple but places a heavy demand on computer memory. Much less computationally intensive is the conjugate gradient method [39], which is now being heavily employed for MOM calculations [40].

Once all ***E***_m_ have been thus calculated, the scattered electric field in *V*_ext_ can be computed as the sum

$$\begin{array}{l} E_{\text{sca}}(x)=E(x)-E_{\text{inc}}(x)=\\ {k_{0}}^{2}\sum_{m\;\in \{1,2,\ldots M\}}\{\nu_{m}[\epsilon_{r,m}-1]\;G(x,x_{m})\cdot E_{m}\};x\in V_{\text{ext},} \end{array}$$ (16)

which expression follows from Eq. (8b). Now, from Eq. (A2-6a), we have the asymptote [41]

$$\begin{array}{l} {\text{Tend}}_{k_{0}\left|x-x_{m}\right|\to \alpha }G(x,x_{m})≅[I-(x-x_{m})(x-x_{m})/\\ {\left|x-x_{m}\right|}^{2}]\left[\exp(ik0\left|x-x_{m}\right|)/4\pi \left|x-x_{m}\right|\right], \end{array}$$ (17a)

correct to order 1/|***x*** *−* ***x****_m_*|. Since all ***x****_m_* are generally distributed around the origin, the further reduction

$$\begin{array}{l} {\text{Tend}}_{k_{0}\left|x-x_{m}\right|\to \infty }G(x,x_{m})≅[I-\mathrm{xx}/{\left|x\right|}^{2}]\\ \{\exp(ik_{0}\left|x-x_{m}\right|)/4\pi \left|x\right|\}, \end{array}$$ (17b)

is reasonable. Finally, making the Fraunhofer approximation [42]

$$\exp\left(ik_{0}\left|x-x_{m}\right|\right)≅\exp\left[ik_{0}|x|\right]\exp\left[ik_{0}x\cdot x_{m}/|x|\right]$$ (17c)

on the left side of Eq. (17b) yields

$$\begin{array}{c} {\text{Tend}}_{k_{0}|x-x_{m}|\to \infty }G\left(x,x_{m}\right)≅\left[I-\mathrm{xx}/{\left|x\right|}^{2}\right]\exp\left(ik_{0}|x|\right)\\ \exp\left(-ik_{0}x\cdot x_{m}/|x|\right)/4\pi |x|]. \end{array}$$ (17d)

Consequently, the scattered electric field of Eq. (16) has the asymptotic representation

$${\text{Tend}}_{k_{0}x\to \infty }E_{\text{sca}}\left(xu_{s}\right)≅[\exp\left(ik_{0}x\right)/x]F_{\text{sca}}\left(u_{s}\right),$$ (18)

in the direction ***u*_s_** in the far zone, with the far-zone scattering amplitude defined by

$$\begin{array}{c} F_{\text{sca}}\left(u_{s}\right)=\left({k_{0}}^{2}/4\pi \right)\left[I-u_{s}u_{s}\right].\\ \sum_{m\;\in \{1,2,..M\}}\left\{\exp\left(-ik_{0}u_{s}\cdot x_{m}\right)\nu_{m}\left[\epsilon_{r,m}-1\right]E_{m}\right\}, \end{array}$$ (19)

which shows quite clearly that ***u*_s_** · ***F*_sca_(*u*_s_) *≡*** 0.

## 5. The Coupled Dipole Method

The heart of the MOM is Eq. (13b) which involves the electric field ***E****_k_* that is *actually present* at ***x****_k_*. However, in the CDM we have to consider the electric field that excites the subregion *V_k_*, each subregion being explicitly modeled as an electric dipole moment. In order to obtain an expression for this exciting field, we return to Eq. (8a), and rewrite it as

$$\begin{array}{c} E\left(x\right)-i\omega \mu_{0}{∭}_{V_{k}}d^{3}x^{\prime }\left\{G\left(x,x^{\prime }\right)\cdot J\left(x^{\prime }\right)\right\}=\\ E_{\text{inc}}\left(x\right)+i\omega \mu_{0}\sum_{m,m\neq k}{∭}_{V_{m}}d^{3}x^{\prime }\left\{G\left(x,x^{\prime }\right)\cdot J\left(x^{\prime }\right)\right\};\\ x\in \sum_{m}V_{m}+V_{\text{ext}}, \end{array}$$ (20)

after partitioning the scatterer region precisely in the same manner as was done for the MOM solution. We know that ***E***_inc_(***x***) is the field in the absence of the scatterer and that the quantity

$$i\omega \mu_{0}{∭}_{V_{m}}d^{3}x^{\prime }\left\{G\left(x,x^{\prime }\right)\cdot J\left(x^{\prime }\right)\right\};x\notin V_{m},m\neq k,$$

is an electric field whose source clearly lies in the subregion *V_m_*, *m ≠ k*. Thus, the whole of the right side of Eq. (20) is a composite electric field with multiple sources, but none of the sources lies in *V_k_*. This composite electric field

$$\begin{array}{c} {\;}^{\left(k\right)}E_{\text{exc}}\left(x\right)=E_{\text{inc}}\left(x\right)+i\omega \mu_{0}\sum_{m,m\neq k}{∭}_{V_{m}}d^{3}x^{\prime }\\ \left\{G\left(x,x^{\prime }\right)\cdot J\left(x^{\prime }\right)\right\};x\in V_{k}, \end{array}$$ (21a)

can be thought of as the field that excites *V_k_*.

Since the left side of Eq. (20) must be equal to its right side, it follows that

$${\;}^{\left(k\right)}E_{\text{exc}}\left(x\right)=E\left(x\right)-i\omega \mu_{0}{∭}_{V_{k}}d^{3}x^{\prime }\left\{G\left(x,x^{\prime }\right)\cdot J\left(x^{\prime }\right)\right\};x\in V_{k}.$$ (21b)

On setting ***x*** = ***x****_k_* in Eq. (21b), and completing the volume integration therein using Eq. (A2-5a), we get the expression

$$\begin{array}{c} E_{\text{exc},k}=E_{k}-i\omega \mu_{0}\left[M_{k}-\left(1/{k_{0}}^{2}\right)L_{k}\right]\cdot J_{k}=E_{\text{exc},k};\\ 1\leq k\leq M; \end{array}$$ (22)

here, ***J****_k_* = ***J***(*x_k_*) and ***E***_exc,_*_k_* = *^(k)^****E***_exc_(***x****_k_*). But, by virtue of Eq. (5c) we have

$$J_{k}=i\omega \epsilon_{0}\left(1-\epsilon_{r,k}\right)E_{k};1\leq k\leq M,$$ (23a)

which means that Eq. (22) reduces to

$$A_{kk}\cdot E_{k}=E_{\text{exc},k};1\leq k\leq M,$$ (23b)

whence,

$$E_{k}={A_{kk}}^{-1}\cdot E_{\text{exc},k};1\leq k\leq M,$$ (23c)

$$J_{k}=i\omega \epsilon_{0}\left(1-\epsilon_{r,k}\right){A_{kk}}^{-1}\cdot E_{\text{exc},k};1\leq k\leq M.$$ (23d)

Consistent with the long-wavelength approach, in the CDM we think of the equivalent electric dipole moment [9, 10]

$$p_{k}=\left(i/\omega \right)\nu_{k}J_{k}$$ (24a)

located at ***x****_k_*; thus,

$$p_{k}=t_{k}\cdot E_{\text{exc},k},$$ (24b)

after using Eq. (21b), where

$$t_{k}=\nu_{k}\epsilon_{0}\left(\epsilon_{r,k}-1\right){A_{kk}}^{-1}$$ (24c)

is the polarizability dyadic of the dielectric region *V_k_*.

We return to Eq. (21a) at this stage, set ***x*** =***x****_k_* therein, and do the volume integrations; the result is the vector dyadic relation

$$\begin{array}{c} E_{\text{exc},k}=E_{\text{inc}}\left(x_{k}\right)+i\omega \mu_{0}\sum_{m,m\neq k}\left\{\nu_{m}G_{km}\cdot J_{m}\right\};\\ 1\leq k\leq M, \end{array}$$ (25)

in accord with the long-wavelength approximation. With the aid of Eq. (24a), we rewrite Eq. (25) as

$$E_{\text{exc},k}=E_{\text{inc}}\left(x_{k}\right)+\omega^{2}\mu_{0}\sum_{m,m\neq k}\left\{G_{km}\cdot p_{m}\right\},$$ (26a)

and the use of Eq. (24b) yields

$$E_{\text{exc},k}=E_{\text{inc}}\left(x_{k}\right)+\omega^{2}\mu_{0}\sum_{m,m\neq k}\left\{G_{km}\cdot t_{m}\right\}\cdot E_{\text{exc},m}.$$ (26b)

Equation (26b) constitutes the core of the CDM and, for numerical work, is best rewritten as the set of the 3*M* algebraic scalar equations

$$E_{\text{inc}}\left(x_{k}\right)=\sum_{m\in \{1,2,\ldots M\}}\left[Q_{km}\cdot E_{\text{exc},m}\right];1\leq k\leq M,$$ (27a)

for the cartesian components of ***E***_exc,_*_m_*, where the dyadic kernel

$$Q_{km}=I\delta_{km}-\omega^{2}\mu_{0}G_{km}\cdot t_{m}\left(1-\delta_{km}\right).$$ (27b)

The CDM algorithm is structurally just the same as the MOM algorithm, as is easily demonstrated by a comparison of Eqs. (27a, b) with Eqs. (14a, b). Hence, computational techniques for solving Eq. (27a) are the same as for solving Eq. (14a).

Once all ***E***_exc,_*_m_* have been obtained as the solution of Eq. (27a), one can use Eq. (24b) to find all ***p****_m_* and ascertain the scattered field as the sum

$$\begin{array}{c} E_{\text{sca}}\left(x\right)=E\left(x\right)-E_{\text{inc}}\left(x\right)=\\ \omega^{2}\mu_{0}\sum_{m\in \{1,2,\ldots M\}}\left\{G\left(x,x_{m}\right)\cdot p_{m}\right\};x\in V_{\text{ext}}, \end{array}$$ (28)

which relation follows from Eq. (8a). Equation (18) still applies for the scattered electric field in the far zone, but the far-zone scattering amplitude is now given by

$$\begin{array}{c} F_{\text{sca}}\left(u_{s}\right)=\left(\omega^{2}\mu_{0}/4\pi \right)\left[I-u_{s}u_{s}\right]\cdot \\ \sum_{m\in \{1,2,\ldots M\}}\left\{\exp\left(-ik_{0}u_{s}\cdot x_{m}\right)p_{m}\right\}; \end{array}$$ (29a)

equivalently,

$$\begin{array}{c} F_{\text{sca}}\left(u_{s}\right)=\left(\omega^{2}\mu_{0}/4\pi \right)\left[I-u_{s}u_{s}\right]\cdot \\ \sum_{m\in \{1,2,\ldots M\}}\left\{\exp\left(-ik_{0}u_{s}\cdot x_{m}\right)t_{m}\cdot E_{\text{exc},m}\right\}. \end{array}$$ (29b)

Parenthetically, we remark here that the Rayleigh-Debye approximation for scattering by smoke agglomerates [11, 12] may be obtained by replacing ***E***_exc,_*_m_* in Eq. (29b) by ***E***_inc_(***x****_m_*).

## 6. Scattering and Absorption

The presence of a scatterer has two observable consequences insofar as the conservation of electromagnetic energy is concerned. A portion of the energy radiated by the source of ***E***_inc_(***x***) is *scattered* in all directions by the scatterer. Another portion is *absorbed* by the scatterer and converted into other forms such as heat. All calculations pertaining to the scattered and the absorbed powers at a given circular frequency *ω* are made in terms of power averaged over the time period 2*π*/*ω*.

Because ***F*_sca_(*u*_s_)** is of the form **[I *− u****_s_****u****_s_***]** · ***b***, it follows that ***u*_s_** · ***F***_sca_(***u*_s_**) ≡ 0, as has been mentioned earlier. By virtue of Eqs. (3a) and (18), we have

$$\begin{array}{c} {\text{Tend}}_{k_{0}x\to \infty }H_{\text{sca}}\left(xu_{s}\right)≅\\ \left(1/\eta_{0}\right)\left[\exp\left(ik_{0}x\right)/x\right]u_{s}\times F_{\text{sca}}\left(u_{s}\right), \end{array}$$ (30)

where *η*_0_ = (*μ*_0_/*ϵ*_0_)^1/2^ is the intrinsic impedance of free space. These observations imply that the scattered field in the far zone is Transverse Electro-Magnetic (TEM) in character.

The time-averaged scattered power per unit solid angle in the direction ***u*_s_** is defined as

$$\begin{array}{c} dP_{\text{sca}}\left(u_{s}\right)/d\Omega \left(u_{s}\right)=\left(1/2\right)\\ {\text{Tend}}_{k_{0}x\to \infty }\text{Real}\left[x^{2}u_{s}\cdot \left\{E_{\text{sca}}\left(xu_{s}\right)\times H_{\text{sca}}*\left(xu_{s}\right)\right\}\right], \end{array}$$ (31a)

with *dΩ*(***u*_s_**) ≡ sin*θ*_s_ d*θ*_s_ d*ϕ*_s_, as is customary in spherical coordinates, and the asterisk denoting the complex conjugate. After putting Eqs. (18) and (30) in Eq. (31a), we obtain

$$dP_{\text{sca}}\left(u_{s}\right)/d\Omega \left(u_{s}\right)=\left(1/2\eta_{0}\right)F_{\text{sca}}\left(u_{s}\right)\cdot F_{\text{sca}}*\left(u_{s}\right);$$ (31b)

consequently, we are able to ascertain the total time-averaged scattered power as the integral

$$\begin{array}{c} P_{\text{sca}}=\left(1/2\eta_{0}\right)\int_{0}^{2\pi }d\phi_{s}\int_{0}^{\pi }d\theta_{s}\sin\theta_{s}\\ F_{\text{sca}}\left(u_{s}\right)\cdot F_{\text{sca}}*\left(u_{s}\right). \end{array}$$ (31c)

Unless the scatterer material is intrinsically lossless, there is absorption of electromagnetic energy in *V*_int_. Because the scatterer is simply a dielectric object here, the time-averaged power absorbed in *V*_int_ may be computed as the volume integral

$$P_{\text{abs}}=\text{Real}\left[\left(i\omega /2\right){∭}_{V_{\mathrm{int}}}d^{3}x\;E\left(x\right)\cdot D*\left(x\right)\right]$$ (32a)

from Poynting’s theorem for monochromatic fields [24, Chap. 2]. After using Eq. (2a) on the right side of this relation, we get the result

$$\begin{array}{c} P_{\text{abs}}=\text{Real}[\left(i\omega /2\right)\\ {∭}_{V_{\mathrm{int}}}d^{3}x\left\{\epsilon_{0}\epsilon_{r}*\left(x\right)E\left(x\right)\cdot E*\left(x\right)\right\}]. \end{array}$$ (32b)

Finally, after using Eq. (12) as well as the long-wavelength approximation made heretofore, we are able to reduce this expression to the sum

$$\begin{array}{c} P_{\text{abs}}=\text{Real}\;[(i\omega /2){∭}_{V_{\mathrm{int}}}d^{3}x\{\epsilon_{0}\epsilon_{r,m}*E_{m}\cdot E_{m}*\}]=\\ -(\omega /2)\;\text{Imag}\;[\sum_{m}\nu_{m}\{\epsilon_{0}\epsilon_{r,m}*E_{m}\cdot E_{m}*\}]. \end{array}$$ (33)

Because ***E****_m_* · ***E***_m_* is purely real, no power absorption will take place in *V_m_* if *ϵ*_r,_*_m_* is purely real; indeed, ***P***_abs_ ≡ 0, provided Imag [*ϵ*_r_(*x*)] ≡ 0 for all *x* ∈ *V*_int_.

Insofar as the MOM is concerned, the solution {***E****_m_*; m = 1, 2,…, *M*} of Eq. (14a) may be directly substituted into Eq. (33) for the computation of ***P***_abs_. The calculation of ***P***_abs_ in the CDM is only slightly more complicated: the exciting fields {***E***_exc,_*_m_*; *m* = 1, 2,…, *M*} obtained by solving Eq. (27a) have to be substituted into Eq. (23c) to get {***E****_m_*;*m* = 1,2,…, *M*}. Thus, an useful expression for CDM computations is

$$\begin{array}{l} P_{\text{abs}}=-(\omega /2)\;\text{Imag}\;[\sum_{m}\nu_{m}\{\epsilon_{0}\epsilon_{r,m}*\\ T_{m}\cdot E_{\text{exc},m}\cdot T_{m}*\cdot E_{\text{exc},m}*\}], \end{array}$$ (34a)

where

$$T_{m}={\left[I+(1-\epsilon_{r,m})\;({k_{0}}^{2}M_{m}-L_{m})\right]}^{-1}.$$ (34b)

The total time-averaged power extinguished by the scatterer region is the sum

$$P_{\text{ext}}=P_{\text{sca}}+P_{\text{abs}}.$$ (35)

Quite often, one is interested in the extinction of the plane wave

$$E_{\text{inc}}(x)=e_{\text{inc}}\exp[ik_{0}u_{\text{inc}}\cdot x],$$ (36a)

$$H_{\text{inc}}(x)=(1/\eta_{0})\;u_{\text{inc}}\times e_{\text{inc}}\exp[ik_{0}u_{\text{inc}}\cdot x],$$ (36b)

where *e*_inc_ carries the units of volts per meter and ***u***_inc_ is a dimensionless unit vector such that ***e***_inc_ · ***u***_inc_ = 0. In this case, the total time-averaged power extinguished by the presence of matter in *V*_int_ can be estimated using the forward scattering amplitude as [22, Chap. 8]:

$$P_{\text{ext}}=(2\pi /\omega \mu_{0})\text{Imag}\;[e_{\text{inc}}*\cdot F_{\text{sca}}(u_{\text{inc}})].$$ (37)

Substitution of Eqs. (29a) and (36a) in this relation gives us

$$P_{\text{ext}}=(\omega /2)\;\text{Imag}\;[\sum_{m}\{E_{\text{inc}}*(x_{m})\cdot p_{m}\}],$$ (38a)

whence,

$$P_{\text{ext}}=(\omega /2)\;\text{Imag}\;[\sum_{m}\{E_{\text{inc}}*(x_{m})\cdot t_{m}\cdot E_{\text{exc},m}\}]$$ (38b)

for use with the CDM. The MOM analog of Eq. (38b) is easily obtained by substituting Eq. (23b) therein; ergo,

$$\begin{array}{c} P_{\text{ext}}=(\omega \epsilon_{0}/2)\;\text{Imag}\;[\sum_{m}\{\nu_{m}(\epsilon_{r,m}-1)\\ E_{\text{inc}}*(x_{m})\cdot E_{m}\}], \end{array}$$ (38c)

for use with the MOM.

## 7. Spherical and Cubical Subrogions

Although spheroidal and ellipsoidal subregions have been used [43, 44], it is commonplace in literature to have cubical or spherical subregions. CDM users are more comfortable with spherical subregions, while MOM users are fond of cubical ones. Cubes and spheres have the same dyadic **L**, and in many MOM codes the **M** dyadic of a cube is estimated as that of an equi-voluminal sphere; see Appendix C. Without any particular loss of generality therefore, we take the subregions to be spherical and of identical radii in the remainder of this paper.

Let the subregion *V_k_* be the sphere of radius *a* with its center at *x_k._* As a result, the volume *v_k_* = (4*π*/3)*a*^3^, the dyadic **L***_k_* = (1/3)**I**, and the dyadic **M***_k_* = (2/3*k*_0_^2^)[(l − *ik*_0_*a*) exp(*ik*_0_*a*) − 1]I, as shown in Appendix C. Consequently, the MOM dyadic kernel given in Eq. (14b) reduces to

$$\begin{array}{c} A_{kk}=I\;[1+(1/3)(1-\epsilon_{r,k})\{2(1-ik_{0}a)\\ \exp(ik_{0}a)-3\}], \end{array}$$ (39a)

$$A_{km}=(4\pi /3)a^{3}{k_{0}}^{2}(1-\epsilon_{r,m})G_{km};m\neq k.$$ (39b)

In the same manner, the CDM dyadic kernel of Eq. (27b) simplifies to

$$Q_{kk}=I,$$ (40a)

$$\begin{array}{l} Q_{km}=-\omega^{2}\mu_{0}(4\pi /3)a^{3}\;\epsilon_{0}(\epsilon_{r,m}-1)\\ \left[1+(1/3)(1-\epsilon_{r,m})\{2(1-ik_{0}a\right)\\ \exp(ik_{0}a)-3\}{]}^{-1}G_{km};m\neq k. \end{array}$$ (40b)

An analysis of the self-terms is crucial to the understanding of the MOM and the CDM. From Eq. (39a) it follows that the MOM self-term can be broken up as

$$A_{kk}=\{{Á}_{kk}+{Å}_{kk}\}I,$$ (41a)

where

$${Á}_{kk}=(\epsilon_{r,k}+2)/3,$$ (41b)

and

$${Å}_{kk}=(2/3)(1-\epsilon_{r,k})[(1-ik_{0}a)\exp(ik_{0}a)-1].$$ (41c)

Both *Á_kk_* and *Å_kk_* should be called self-terms; instead, it seems only *Å_kk_* has been accorded that honor in some MOM papers, e.g., [16–19, 45].

The CDM self-term is somewhat obscure, being hidden as the polarizability dyadic **t***_k_* of Eq. (24b). In the present instance, the polarizability dyadic reduces to a scalar because the subregion is spherical; hence,

$$t_{k}=I\;\tau_{k},$$ (42a)

where

$$\tau_{k}=\alpha_{k}/(1+{Å}_{kk}/{Á}_{kk}),$$ (42b)

and

$$\alpha_{k}=4\pi a^{3}\epsilon_{0}(\epsilon_{r,k}-1)/(\epsilon_{r,k}+2)$$ (42c)

is the Mossotti-Clausius polarizability [46, 47] of the electrically small dielectric sphere.

Let *k*_0_*a* < < 1 in Eq. (41c) and *Å_kk_* be evaluated correct to order *k*_0_^3^*a*^3^. Then,

$$\tau_{k}≅\alpha_{k}/\{1-{k_{0}}^{2}(a^{-1}+2ik_{0}/3)\alpha_{k}/4\pi \epsilon_{0}\},$$ (43)

and we observe that the (2*ik*_0_^3^/3)*α*_k_/4*πϵ*_0_ term in the denominator of the right side of Eq. (43) is the radiative reaction term used by Draine [48] in his CDM formulation. More often, *Å_kk_* is evaluated correct only to the first order in *k*_0_*a*, leading to *τ_k_* ≅ *α_k_* [7], and thereby giving rise to the semi-microscopic flavor of this numerical approach [9].

## 8. Strong and Weak Forms

The various approximations that can be made for the self-terms lead us to the *strong* and the *weak* forms of the MOM and the CDM [10]. In the strong forms (S-CDM & S-MOM), Eq. (A2-5a) is used to estimate the self-term (*m = k*) in Eqs. (13a) and (21b). In the Weak forms (W-CDM & W-MOM), Eq. (A2-7) is used in place of Eq. (A2-5a) for this estimation. For isotropic dielectric scatterers, the W-CDM is exemplified by Purcell and Pennypacker [7] using spherical subregions, and the S-CDM has become recently available [17] for the same problem. The S-MOM has been used for isotropic dielectric scatterers for many years [6, 33], but the idea of W-MOM is of more recent provenance [17].

The W-MOM corresponds exactly to the W-CDM, while the S-MOM corresponds exactly to the S-CDM. When all subregional volumetric capacities are very small, the S-MOM/S-CDM effectively transmutes into the W-MOM/W-CDM. Generally stated, therefore, it can be surmised that the scatterer region *V*_int_ must be broken up into a larger number of subregions when the W-MOM/W-CDM is used than if the S-MOM/S-CDM is used. Comparison of S-MOM results with the W-CDM results, with identical partitioning of the scatterer region, [e.g., 16, 18, 19], in some instances may be like comparing apples with oranges. The difference between S-CDM/S-MOM and W-CDM/W-MOM is solely due to the inclusion or the exclusion of the dyadic **M***_k_* in the expression for **A***_kk_*, therefore computational time is marginally increased and the memory requirement negligibly, when one shifts from W-CDM/W-MOM to S-CDM/S-MOM.

## 9. Spherical Subregions and Finite-Size Effects

An assessment of the MOM and the CDM for isotropic dielectric scatterers with spherical subregions is now in order. To facilitate such a comparison, we reiterate that

$$\begin{array}{cc} A_{kk}=\{1+(1+\epsilon_{r,k})[(2/3)(1-ik_{0}a) & \\ \exp(ik_{0}a)-1]\}I, & \left[\text{S-MOM}\right] \end{array}$$ (44a)

$$\begin{array}{cc} A_{kk}≅\{(\epsilon_{r,k}+2)/3\}I; & \left[\text{W-MOM}\right] \end{array}$$ (44b)

and correspondingly,

$$\begin{array}{cc} \tau_{k}=(4\pi /3)a^{3}\epsilon_{0}(\epsilon_{r,k}-1)/\{1+(1-\epsilon_{r,k})[(2/3) & \\ (1-ik_{0}a)\;\exp(ik_{0}a)-1]\}, & \left[\text{S-CDM}\right] \end{array}$$ (45a)

$$\begin{array}{cc} \tau_{k}≅4\pi a^{3}\epsilon_{0}(\epsilon_{r,k}-1)/(\epsilon_{r,k}+2)=\alpha_{k}. & \\ & \left[\text{W-CDM}\right] \end{array}$$ (45b)

We can evaluate the strong forms vis-a-vis the weak forms through the Taylor expansion of the ratio r/a about *ϵ_r_* = 1, there being no need for us to continue the subscript *k* in the remainder of this paper. Using Eqs. (41b), (41c) and (42b), we get the expansion

$$\begin{array}{l} \tau /\alpha =1+2\Lambda \left[\left(\epsilon_{\text{r}}-1\right)/3\right]+\\ 2\Lambda \left(2\Lambda -1\right){\left[\left(\epsilon_{\text{r}}-1\right)/3\right]}^{2}+\cdots , \end{array}$$ (46a)

where *Λ* = [(1 *− ik*_0_*a*) exp(*ik*_0_*a*) *−* 1] has been used for convenience; and it becomes clear that the weak forms are poor approximations of the strong forms when the relative permittivity is significantly different from unity.

Likewise, the Maclaurin expansion of *τ*/*α* with respect to the size parameter *k_0_a*,

$$\begin{array}{l} \tau /\alpha =1+[(\epsilon_{r}-1)/(\epsilon_{r}+2)]{\left(k_{0}a\right)}^{2}+\\ +(2i/3)[(\epsilon_{r}-1)/(\epsilon_{r}+2)]{\left(k_{0}a\right)}^{3}+\ldots , \end{array}$$ (46b)

indicates that the weak forms become increasingly inadequate as the size parameter of the subregion increases.

**Fig. 2:**
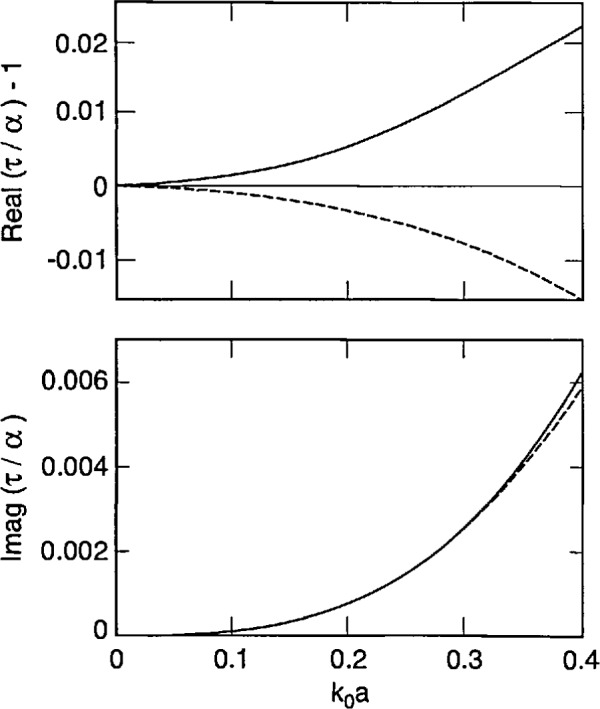
Functions [*τ*/*α* − 1] (solid lines) and [*τ*_DB_/*α* − 1] (dashed lines) as functions of the size parameter *k*_0_*a* of a spherical sub-region whose relative permittivity *ϵ*_r_ = 1.5; the Mossotti-Clausius polarizability *α* = 4*πa*^3^ *ϵ*_0_(*ϵ*_r_ − 1)/(*ϵ*_r_ *+* 2).

**Fig. 3:**
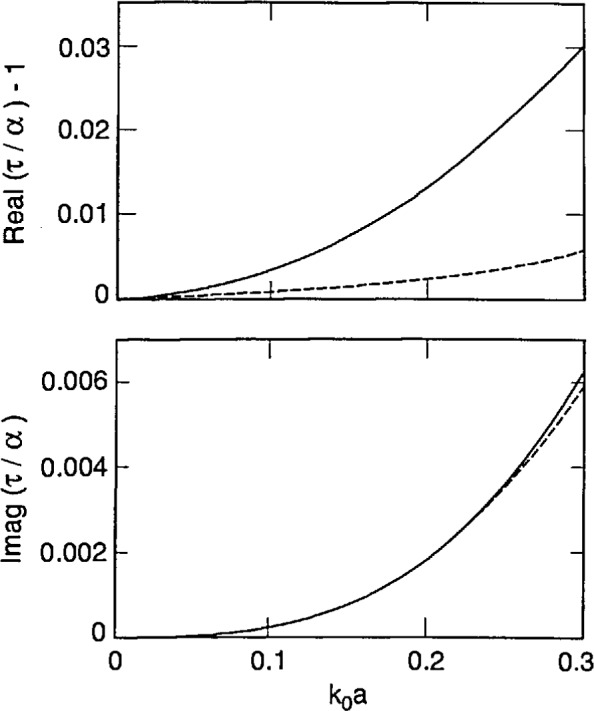
Same as Fig. 2, but *ϵ*_r_ = 2.5.

**Fig. 4:**
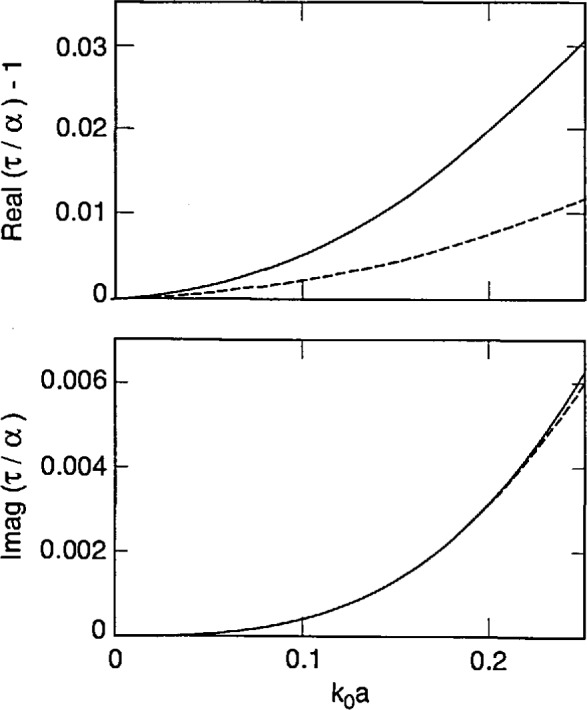
Same as Fig. 2, but *ϵ*_r_ = 4.0.

**Fig. 5:**
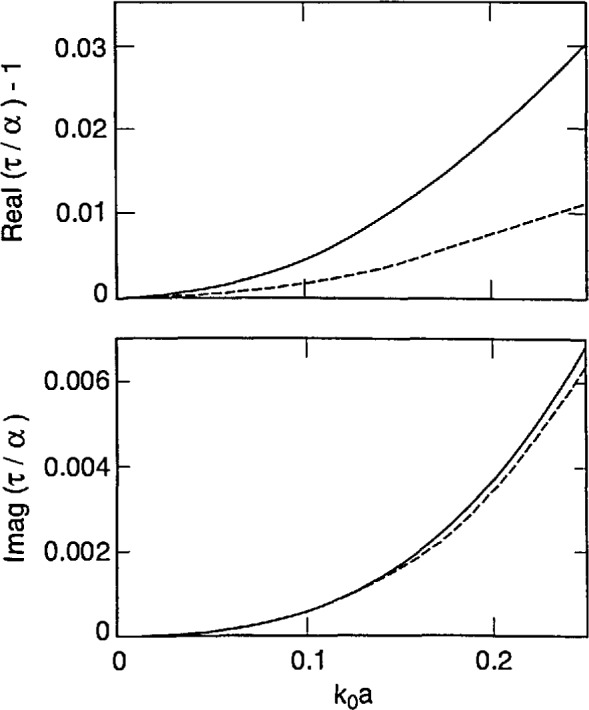
Same as Fig. 2, but *ϵ*_r_ = 3.75 + 0.25*i*.

Equations (46a) and (46b) tell us that strong forms incorporate finite-size effects meaningfully, while the weak forms do so trivially. This conclusion is reinforced by Figs. 2–4 that show plots of *τ*/*α* versus the normalized radius *k*_0_*a* of an electrically small sphere for *ϵ_r_* = 1.5, 2.5 and 4.0. In these figures we have ensured that the maximum value of *k*_0_*a|ϵ_r_*^1/2^| is about 0.5. We observe – not surprisingly – that *τ* is complex while *α* is purely real for these input parameters. Furthermore, in all three figures the ratio |*τ*/*α*| lies between 1.02 and 1.03 when *k*_0_*a|ϵ_r_*^1/2^| ≅ 0.5; while *τ*/α = 1 for *k*_0_*a* = 0, of course. Similar conclusions can be drawn from Fig. 5, wherein the calculations of *τ*/α have been reported for a lossy dielectric sphere with *ϵ*_r_ = 3.75 + 0.25*i*. These figures highlight the understanding on finite-size effects drawn from Eqs. (46a, b), and it follows that coarser partitioning of *V*_int_ may be acceptable when S-CDM/S-MOM is used than when W-CDM/W-MOM is used. These thoughts can be validated by careful examination of the graphs recently published by Ku [19].

The strong forms discussed above are not the only way of introducing finite-size effects, there being at least two more CDM algorithms available for that purpose. As alluded to earlier, Draine [48] replaced the Mossotti-Clausius polarizability by

$$\begin{array}{cc} \tau_{D}=\alpha /\{1-(i/6\pi \epsilon_{0}){k_{0}}^{3}\alpha \}. & \left[\text{D-CDM}\right] \end{array}$$ (47)

However, *τ*_D_ is fully contained in *τ*, as we have seen in Eq. (43); and *τ*_D_ approaches *α* as the size parameter *k*_0_*a* becomes vanishingly small.

The second source for the incorporation of finite-size effects has been the Lorentz-Mie-Debye analysis for scattering by a dielectric sphere [46]. The electromagnetic field scattered by any object can be described in terms of a multipole expansion [49]. With inspiration from Doyle [50], Dungey and Bohren [51] introduced finite-size effects in the CDM by using the lowest order Mie coefficient – corresponding to the electric dipole term of the mutipole expansion – for the polarizability in place of the Mossotti-Clausius polarizability *α*. Thus,

$$\begin{array}{l} \tau_{\text{DB}}=(i6\pi \epsilon_{0}/{k_{0}}^{3})\\ \frac{\left[{\epsilon_{r}}^{1/2}\psi (k_{0}a{\epsilon_{r}}^{1/2})\partial \psi (k_{0}a)-\psi (k_{0}a)\partial \psi (k_{0}a{\epsilon_{r}}^{1/2})\right]}{\left[{\epsilon_{r}}^{1/2}\psi (k_{0}a{\epsilon_{r}}^{1/2})\partial \zeta (k_{0}a)-\zeta (k_{0}a)\partial \psi (k_{0}a{\epsilon_{r}}^{1/2})\right]}\;[\text{DB-CDM}] \end{array}$$ (48)

was used in [47], with *ψ*(*β*) *= β*^−1^ sin(*β*) − cos(*β*), ∂*ψ*(*β*) = d*ψ*/df/d*β*, *ζ*(*β*) = − (1 + i/*β*) exp(*iβ*), and ∂*ζ*(*β*) = d*ζ*/*dβ*.

Using Eq. (48), it can be shown that *τ*_DB_ approaches *α* as the normalized radius *k*_0_*a* becomes vanishingly small. This is also borne out in Figs. 2–5, wherein the computed values of *τ*_DB_/*α* are compared with those of *τ*/*α* as functions of the normalized radius *k*_0_*a* for *ϵ*_r_ = 1.5, 2.5, 4.0 and 3.75 + 0.2*i*. The general behavior of *τ*_DB_ is the same as of *τ*: (i) *τ*_DB_ is complex even for purely real *ϵ*_r_ and (ii) the ratio |*τ*_DB_/*α*| lies between 0.99 and 1.01 when *k*_0_*a |ϵ*_r_^1/2^| ≅ 0.5 in these figures. A more remarkable observation is that |1 − *τ*_DB_/*α*| ≤ |1 − *τ*/*α*|; in other words, *τ*_DB_ is closer to the Mossotti-Clausius polarizability *α* than *τ* is.

Draine [48] and Dungey and Bohren [51] concluded from their numerical investigations that D-CDM and DB-CDM, respectively, generally provide scattering results superior to those from W-CDM. This does not come as a surprise because the self-terms in W-CDM (or W-MOM) are estimated with the least accuracy. On the other hand, although it is difficult to provide general enough conclusions for the adequacy of either D-CDM or DB-CDM vis-a-vis that of the S-CDM/S-MOM, it is safe to state that any claims of superiority – based purely on the estimation of some gross parameter, such as the total scattering cross-section – are debatable. Indeed, the only good way of deciding on the superiority of S-CDM, D-CDM or DB-CDM is by making calculations for the specific problem under consideration: The scatterer region should be subdivided into different numbers of subregions, and computed data from the various algorithms for different partitioning schemes compared for the property of interest [52–54].

**Fig. 6:**
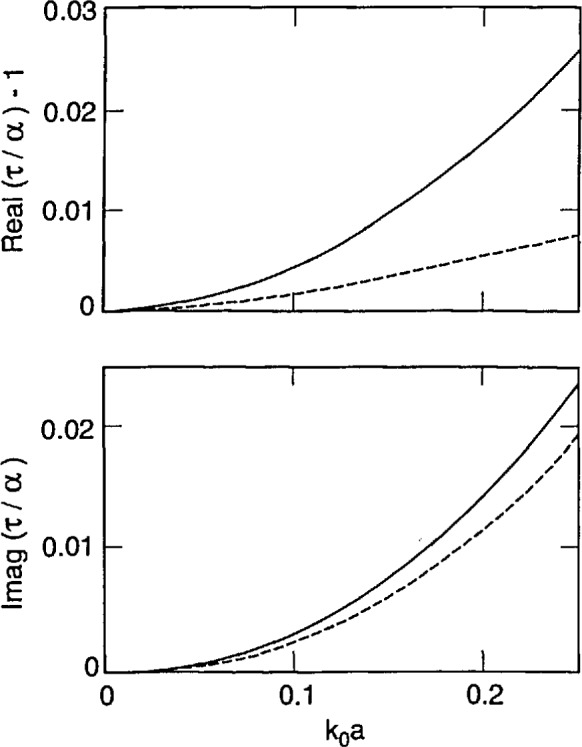
Same as Fig. 2, but *ϵ*_r_ = 2.40 + 2.38*i*.

The refractive index of soot at visible frequencies has been measured by a variety of experimental techniques, and has been found to be dependent on the source materials [55]. We conclude with calculations of *τ*/*α* and *τ*_DB_/*α* for *ϵ*_r_ = 2.40 + 2.38*i*, corresponding to a complex refractive index of 1.7 + 0.7*i*, in Fig. 6. It is not difficult to gather from this figure that conclusions drawn in the previous paragraphs of this section apply to light scattering by soot agglomerates.

## Figures and Tables

**Fig. 7:**
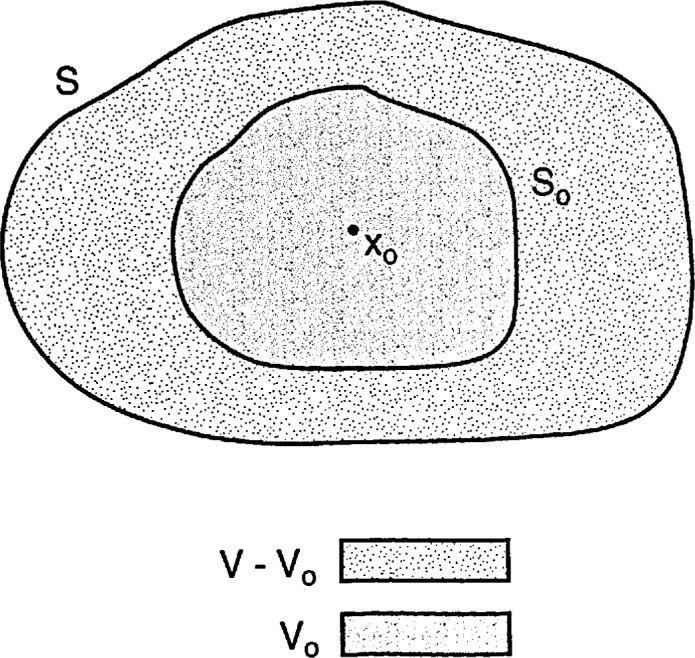
For the evaluation of the integral in Eq. (A2-1) when *x*_0_ ∈ *V* and *x*′ ∈ *V.*

## 10. Appendix A. The Volume Integral Equation for the Electric Field

The free space Green's function **G**(***x,x*′**) satisfies the dyadic differential equation

$$\begin{array}{c} \nabla \times \nabla \times G(x,x^{\prime })-{k_{0}}^{2}G(x,x^{\prime })=\\ I\;\delta (x,-x^{\prime }), \end{array}$$ (A1-1)

where *δ*(*x−x'*) is the Dirac delta function. Since *J*(*x*′) is not a function of *x*, it follows that *∇* × [**G**(*x*,*x*′) · ***J***(*x*′)] = [*∇* × **G**(*x*, *x*′)] · *J*(*x*′); hence,

$$\begin{array}{c} \nabla \times \nabla \times [G(x,x^{\prime })\cdot J(x^{\prime })]-\\ {k_{0}}^{2}[G(x,x^{\prime })\cdot J(x^{\prime })]=I\cdot J(x^{\prime })\;\delta (x-x^{\prime }). \end{array}$$ (A1-2)

We multiply both sides of this equation by *iωμ*_0_ and integrate over all *x*′ to get

$$\begin{array}{c} (\nabla \times \nabla \times I-{k_{0}}^{2}I)\cdot {∭}_{\left|x^{\prime }\right|\leq \infty }d^{3}x^{\prime }\\ {}[i\omega \mu_{0}\;G(x,x^{\prime })\cdot J(x^{\prime })]=\\ i\omega \mu_{0}{∭}_{\left|x^{\prime }\right|\leq \infty }d^{3}x^{\prime }\;J(x^{\prime })\;\delta (x,x^{\prime }), \end{array}$$ (A1-3)

whence,

$$\begin{array}{c} (\nabla \times \nabla \times I-{k_{0}}^{2}I)\cdot {∭}_{\left|x^{\prime }\right|\leq \infty }d^{3}x^{\prime }\\ {}[i\omega \mu_{0}\;G(x,x^{\prime })\cdot J(x^{\prime })]=i\omega \mu_{0}J(x). \end{array}$$ (A1-4)

Now by comparing Eqs. (7) and (A1-4), it is not too arduous to convince ourselves that

$$\begin{array}{c} E(x)=i\omega \mu_{0}{∭}_{\left|x^{\prime }\right|\leq \infty }d^{3}x^{\prime }\;[G(x,x^{\prime })\cdot J(x^{\prime })];\\ 0\leq \left|x\right|\leq \infty . \end{array}$$ (A1-5)

Remembering that for the present purposes ***J*(*x*)** is null everywhere except in *V*_int_, we next obtain

$$\begin{array}{c} E(x)=i\omega \mu_{0}{∭}_{V_{\mathrm{int}}}d^{3}x^{\prime }\;[G(x,x^{\prime })\cdot J(x^{\prime })];\\ x\in V_{\mathrm{int}}+V_{\text{ext}}. \end{array}$$ (A1-6)

The solution of every linear differential equation can be divided into two parts: the *particular solution* that depends on the *forcing function*, ***J*(*x*)** being the forcing function here, and the *complementary function* that holds when the forcing function is identically zero everywhere. The right side of Eq. (A1-6) is the particular solution of Eq. (7), which has the complementary function *E*_inc_(*x*) satisfying

$$\begin{array}{c} \nabla \times \nabla \times E_{\text{inc}}(x)-{k_{0}}^{2}E_{\text{inc}}(x)=0;\\ x\in V_{\mathrm{int}}+V_{\text{ext}}. \end{array}$$ (A1-7)

Hence, the complete solution of Eq. (7) is

$$\begin{array}{c} E(x)=E_{\text{inc}}(x)+i\omega \mu_{0}{∭}_{V_{\mathrm{int}}}d^{3}x^{\prime }\\ {}[G(x,x^{\prime })\cdot J(x^{\prime })];\;x\in V_{\mathrm{int}}+V_{\text{ext}}, \end{array}$$ (A1-8)

which can be easily re-expressed as Eq. (8a).

## 11. Appendix B. The Self-Integral

The integral under consideration is given as

$$a(x_{0})={∭}_{V}d^{3}x^{\prime }\;G(x_{0},x^{\prime })\cdot b(x^{\prime }),$$ (A2-1)

where *V* is the region bounded by the surface *S*, as shown in Fig. 7, **G(*x***, ***x*′**) is given by Eq. (8c), and the point *x*_0_ lies inside *V*. Because **G(*x***, ***x*′**) is of the order 1/|*x* − *x*′|^3^ for |*x* − *x′*| ≅ 0, and becomes singular at *x = x*′, this integral has to be carefully treated.

As shown by Fikioris [56], we can transform Eq. (A2-1) to

$$\begin{array}{c} a(x_{0})={∭}_{V-V_{0}}d^{3}x^{\prime }\;[G(x_{0},x^{\prime })\cdot b(x^{\prime })]+\\ {∭}_{V_{0}}d^{3}x^{\prime }\;[G(x_{0},x^{\prime })\cdot b(x^{\prime })-G_{s}(x_{0},x^{\prime })\cdot b(x_{0})]\\ -(1/{k_{0}}^{2})\iint_{S_{0}}d^{2}x^{\prime }\;{u_{n}}^{\prime }\\ \left[(x^{\prime }-x_{0})/4\pi {\left|x^{\prime }-x_{0}\right|}^{3}\right]\cdot b(x_{0}), \end{array}$$ (A2-2)

where

$$G_{s}(x,x^{\prime })=(1/{k_{0}}^{2})\;\nabla \nabla [1/4\pi \left|x-x^{\prime }\right|]$$ (A2-3)

is an auxiliary dyadic related to the Green’s function for Poisson’s equation [10]; *V*_0_ is an exclusionary region bounded by the surface *S*_0_, as shown in Fig. 7; *u_n_* is the unit normal to *S*_0_, pointing away from *V*_0_, at the point *x*′ ∈ *S*_0_. The exclusionary region *V*_0_ should be small but not necessarily infinitesimal, and it must be wholly contained within *V*. Moreover, there is no requirement that *S*_0_ be a miniature copy of *S*.

The use of the long-wavelength approximation *b*(*x*)≅*b*(*x*_0_) for all *x* ∈ *V* reduces Eq. (A2-2) to

$$\begin{array}{c} a(x_{0})≅{∭}_{V-V_{0}}d^{3}x^{\prime }\;[G(x_{0},x^{\prime })\cdot b(x_{0})]+\\ {∭}_{V_{0}}d^{3}x^{\prime }\;\{[G(x_{0},x^{\prime })-G_{s}(x_{0},x^{\prime })]\cdot b(x_{0})\}\\ -(1/{k_{0}}^{2})\iint_{S_{0}}d^{2}x^{\prime }\;{u_{n}}^{\prime }[(x^{\prime }-x_{0})/\\ 4\pi {\left|x^{\prime }-x_{0}\right|}^{3}]\cdot b(x_{0}). \end{array}$$ (A2-4)

Since *V* is electrically small, but *V*_0_ need not be infinitesimal, we set *V = V*_0_ and *S = S*_0_ to have

$$a(x_{0})≅[M-(1/{k_{0}}^{2})L]\cdot b(x_{0}),$$ (A2-5a)

where

$$M={∭}_{V}d^{3}x^{\prime }\;[G(x_{0},x^{\prime })-G_{s}(x_{0},x^{\prime })],$$ (A2-5b)

$$L=\iint_{S}d^{2}x^{\prime }\;{u_{n}}^{\prime }[(x^{\prime }-x_{0})/4\pi {\left|x^{\prime }-x_{0}\right|}^{3}].$$ (A2-5c)

The evaluation of **M** can be accomplished numerically for regions of different shapes using the coordinate-free (i.e., non-indicial) representations [24, Chap. 9]

$$\begin{array}{c} G(x,x^{\prime })=[(I-u_{X}u_{X})+(i/k_{0}\left|X\right|)(1+i/k_{0}\left|X\right|)\\ (I-3\;u_{X}u_{X})]\;[\exp\;(ik_{0}\left|x-x^{\prime }\right|)/4\pi \left|x-x^{\prime }\right|];\\ \left|X\right|\neq 0, \end{array}$$ (A2-6a)

$$\begin{array}{c} G_{s}(x,x^{\prime })={\left(i/k_{0}\left|X\right|\right)}^{2}\left(I-3\;u_{X}u_{X}\right)]/4\pi \left|X\right|;\\ \left|X\right|\neq 0, \end{array}$$ (A2-6b)

where ***X = x−x*′** and ***u****_x_* ***= X/|X*|.** The evaluation of the depolarization dyadic **L** is equally easy on a digital computer, and analytical expressions for **L** are available for the quite general ellipsoidal shapes [57].

If *V* is extremely small in electrical size, the simplification

$$a(x_{0})≅(1/{k_{0}}^{2})\;L\cdot b(x_{0})$$ (A2-7)

is permissible and leads to the weak forms of the MOM and the CDM.

## 12. Appendix C: The Self-Dyadics for Spherical and Cubical Regions

Let the region *V* in Appendix B be a sphere with radius *a* and center at *x*_0_. Without loss of generality due to the translational invariance of the right sides of Eqs. (A2-6a, b), we set ***x*_0_*= 0***. Furthermore, we set *x′* = r′ (*u*_1_ sin*θ*′ cos*ϕ*′ + *u*_2_ sin*θ*′ sin*ϕ*′ + *u*_3_ cos*θ*′), where |*x′*| = r′, (*u*_1_, *u*_2_, *u*_3_) is the triad of cartesian unit vectors, while (r′, *θ*′, *ϕ*′) represent the corresponding spherical coordinate system located at the center of the sphere. Hence,

$$\begin{array}{c} {∭}_{V}d^{3}x^{\prime }\{\cdot \}\equiv \int_{0}^{a}dr^{\prime }\;{r^{\prime }}^{2}\int_{0}^{\pi }d\theta^{\prime }\;\sin\theta^{\prime }\int_{0}^{2\pi }d\phi^{\prime }\{\cdot \},\\ \iint_{S}d^{2}x^{\prime }\{\cdot \}\equiv a^{2}\int_{0}^{\pi }d\theta^{\prime }\;\sin\theta^{\prime }\int_{0}^{2\pi }d\phi^{\prime }\{\cdot \}. \end{array}$$

Now,

$$\begin{array}{c} \int_{0}^{\pi }d\theta^{\prime }\;\sin\theta^{\prime }\;\int_{0}^{2\pi }d\phi \;u_{x}u_{x}=\\ \int_{0}^{\pi }d\theta^{\prime }\;\sin\theta^{\prime }\;\int_{0}^{2\pi }d\phi^{\prime }\;\{(u_{1}\sin\theta^{\prime }\;\cos\phi^{\prime }+\\ u_{2}\sin\theta^{\prime }\;\sin\phi^{\prime }+u_{3}\cos\theta^{\prime })(u_{1}\sin\theta^{\prime }\;\cos\phi^{\prime }+u_{2}\\ \sin\theta^{\prime }\;\sin\phi^{\prime }+u_{3}\cos\theta^{\prime })\}=\\ \pi \int_{0}^{\pi }d\theta^{\prime }\;\sin\theta^{\prime }\{(u_{1}u_{1}+u_{2}u_{2})\sin^{2}\theta +2u_{3}u_{3}\cos^{2}\theta^{\prime }\}=\\ (4\pi /3)(u_{1}u_{1}+u_{2}u_{2}+u_{3}u_{3})=(4\pi /3)I, \end{array}$$ (A3-1)

and

$$\int_{0}^{\pi }d\theta^{\prime }\;\sin\theta^{\prime }\int_{0}^{2\pi }d\phi^{\prime }\;I=4\pi I$$ (A3-2)

likewise. It follows that

$$\begin{array}{c} M{|}_{\text{sphere}}=\int_{0}^{a}dr^{\prime }\;{r^{\prime }}^{2}\int_{0}^{\pi }d\theta^{\prime }\;\sin\theta^{\prime }\int_{0}^{2\pi }d\phi^{\prime }\\ \left\{[G(0,x^{\prime })-G_{s}(0,x^{\prime })]\right\}\\ =\int_{0}^{a}dr^{\prime }\;{r^{\prime }}^{2}\;[(2I/3)\;\exp(ik_{0}r^{\prime })/r^{\prime }]\\ =I(2/3{k_{0}}^{2})\;[(1-ik_{0}a)\exp(ik_{0}a)-1]. \end{array}$$ (A3-3)

In order to evaluate **L** for a spherical region, we note that *u_n_′ = x*′/|*x′*| where *x*′ ∈ *S*; ergo, |*x′| = a*, and

$$\begin{array}{c} L{|}_{\text{sphere}}=a^{2}\int_{0}^{\pi }d\theta^{\prime }\;\sin\theta^{\prime }\;\int_{0}^{2\pi }d\phi^{\prime }\\ \left\{{u_{n}}^{\prime }x^{\prime }/4\pi a^{3}\}=(1/4\pi \right)\\ \int_{0}^{\pi }d\theta^{\prime }\;\sin\theta^{\prime }\int_{0}^{2\pi }d\phi^{\prime }\;\{(u_{1}\;\sin\theta^{\prime }\;\cos\phi^{\prime }+\\ u_{2}\;\sin\theta^{\prime }\;\sin\phi^{\prime }+u_{3}\;\cos\theta^{\prime })(u_{1}\;\sin\theta^{\prime }\;\cos\phi^{\prime }+\\ u_{2}\;\sin\theta^{\prime }\;\sin\phi^{\prime }+u_{3}\;\cos\theta^{\prime })\}\\ =(1/4\pi )\;(4\pi /3)I=(1/3)I, \end{array}$$ (A3-4)

after using Eq. (A3-1).

If the region *V* in Appendix B were to be a cube of side *b*, we would get

$$L{|}_{\text{cube}}=(1/3)I,$$ (A3-5)

the same as that for a sphere. No expression exists for the dyadic **M** for a cube in closed form; however, it is commonplace in MOM practice to use the value for an equivoluminal sphere. Thus,

$$\begin{array}{c} M{|}_{\text{cube}}≅I(2/3{k_{0}}^{2})\;\{[1-ik_{0}b{\;(3/4\pi )}^{1/3}]\\ \exp[ik_{0}b\;{\left(3/4\right)}^{1/3}]-1\}, \end{array}$$ (A3-6)

because [(3/4*π*)^1/3^ *b*] is the radius of a sphere having the same volume as the cube of side *b*.

## Glossary

*0*: Null vector
**0**: Null dyadic
*a*: Radius of a spherical subregion
*a*, *b*, *d*_1_, *d*_2_: Arbitrary vectors
*a_m_*: (1/2) × maximum linear cross-sectional dimension of the sub-region *V_m_*
A*_km_*: Dyadic kernel for the MOM equations
*Å_kk_*, *Á_kk_*: Self-terms when MOM is used with spherical subregions
*b*: Side of a cubical subregion
*B*(*x*): Magnetic flux density (weber per square meter)
*D*(*x*): Electric flux density (coulomb per square meter)
*e*_inc_: Electric field intensity amplitude of an incident plane wave
*E*(*x*): Electric field intensity (volt per meter)
*^(m)^E*_exc_(*x*): Electric field intensity exciting the subregion *V_m_*
*E*_cxc,m_: = ^(^*^m^*^)^*E*_exc_(*X_m_*)
*E*_inc_(*x*): Incident electric field intensity
*E_m_*: Electric field intensity at *x_m_*
*E*_sca_(*x*): Scattered electric field intensity
*F*_sca_(*u*_s_): Far-zone scattering amplitude in the direction *u_s_*
G(*x*, *x*′): Free space dyadic Green’s function
G*_km_*: = G(*x_k_*, *x_m_*)
*H*(*x*): Magnetic field intensity (ampere per meter)
*H*_sca_(*x*): Scattered magnetic field intensity
*i*: = (−1)^1/2^
I: Identity dyadic
*J*(*x*): Electric current density (ampere per cubic meter)
*J_m_*: = *J*(*x_m_*)
*J*_cond_(*x*): Conduction current density (ampere per cubic meter)
*J*_pol_(*x*): Polarization current density (ampere per cubic meter)
*k*_0_: Free space wavenumber (inverse meter)
**L***_m_*, **M***_m_*, **T***_m_*: Depolarization dyadics for the three-dimensional subregion *V_m_*
*M*: Total number of subregions
*p_m_*: Electric dipole moment equivalent to the subregion *V_m_*
***P***_abs_: Time-averaged absorbed power
***P***_sca_: Time-averaged scattered power
***P***_ext_: Time-averaged extinguished power
**Q***_km_*: Dyadic kernel for the CDM equations
***S****_m_*: Closed surface of the three-dimensional subregion *V_m_*
***t***: Time (seconds)
**t***_m_*: Polarizability dyadic of the subregion *V_m_*
***u*_s_**: Unit vector denoting direction in the far zone
***u***_inc_: Unit vector denoting propagation direction of an incident plane wave
*V*_ext_: Three-dimensional region external to the scatterer
*V*_int_: Three-dimensional region occupied by the scatterer
*V_m_*: Three-dimensional subregion
*x*: Three-dimensional position vector (meter)
*x_m_*: Distinguished point inside the subregion *V_m_*
***X****_km_*: *x_k_*-*x_m_*
*α*: Mossotti-Clausius polarizability scalar of a spherical subregion
*α_m_*: Mossotti-Clausius polarizability scalar of the *m*-th spherical subregion
*δ_km_*: Kronecker delta
*δ*(*x–x*′): Dirac delta
ϵ_0_: Permittivity of free space (= 8.854 × 10^−12^ farad per meter)
ϵ_r_: Relative permittivity
ϵ_r_(*x*): Relative permittivity of dielectric matter occupying *V*_int_
ϵ_r_, *_m_*: Relative permittivity of dielectric matter of the subregion *V_m_*
*η*_0_: Intrinsic impedance of free space (= 120 π ohm)
*Λ*: = [(1 − *ik*_0_*a*) exp(*ik*_0_*a*) − 1]
*λ*: = Wavelength in free space (= 2π/*k*_0_)
*μ*_0_: Permeability of free space (= 4*π* × 10^−7^
*ω*: Circular frequency (radian per second)
*Ω*: Solid angle
*τ*: Polarizability scalar of a spherical subregion
*τ*_m_: Polarizability scalar of the *m*-th spherical subregion
*τ*_D_: Draine’s polarizability scalar of a spherical subregion
*τ*_DB_: Dungey-Bohren polarizability scalar of a spherical subregion
*ν_m_*: Volume of the subregion *V_m_* (cubic meter)
*ψ*(*β*): A Riccati-Bessel function of argument *β*
*ζ*(*β*): A Riccati-Hankel function of argument *β*
